# Detecting downhole vibrations through drilling horizontal sections: machine learning study

**DOI:** 10.1038/s41598-023-33411-9

**Published:** 2023-04-17

**Authors:** Ramy Saadeldin, Hany Gamal, Salaheldin Elkatatny

**Affiliations:** Department of Petroleum Engineering, King Fahd University of Petroleum and Minerals, Box: 5049, Dhahran, 31261 Saudi Arabia

**Keywords:** Energy science and technology, Engineering

## Abstract

During the drilling operations and because of the harsh downhole drilling environment, the drill string suffered from downhole vibrations that affect the drilling operation and equipment. This problem is greatly affecting the downhole tools (wear and tear), hole problems (wash-out), mechanical energy loss, and ineffective drilling performance. Extra non-productive time to address these complications during the operation, and hence, extra cost. Detecting the drillstring vibrations during drilling through the downhole sensors is costly due to the extra service and downhole sensors. Currently, the new-technology-based solutions are providing huge capabilities to deal intelligently with the data, and machine learning applications provide high computational competencies to learn and correlate the parameters for technical complex problems. This research presented a successful case study for developing machine learning models through a comprehensive methodology process for vibration detection using surface rig data through data collection, preprocessing, analytics, training and optimizing the models’ parameters, and evaluating the performance to have the best prediction results. Evaluating the models’ performance showed that obtained predictions have a great match with actual measurements for the different stages of training, testing, and even during models’ validation with unseen well data. Real-field horizontal drilling data was utilized to feed and train the models through different tools named radial basis function (RBF), support vector machines (SVMs), adaptive neuro-fuzzy inference system (ANFIS), and functional networks (FN) to auto-detect the three types of downhole vibrations (axial, torsional, and lateral). The study results showed a high correlation coefficient (higher than 0.9) and technically accepted average absolute percentage error (below 7.5%) between actual readings and predictions of the developed ML models. The study outcomes will add to the automation process of drilling operations to avoid many tools failure by comparing predicted vibrations versus downhole tools limits such as red zone and continuing drilling without interruption to the well total depth especially while drilling horizontal sections.

## Introduction

Drilling performance monitoring is a critical role for the drilling team to be aware of the operation situation and be ready to take the correct actions for safe operations and save extra cost for the nonproductive time due to any drilling problems. The downhole drill string vibration is a critical issue during the drilling operation and many drilling problems can be associated with the extraordinary level of vibrations as wear and tear for the drill bit and drillstring, inefficient performance for the drilling operation, power and cost losses, drilled hole instability issues^1,2^, besides equipment failure due to existing stresses and deformation^3^. Consequently, close monitoring of the downhole drillstring vibration will help to make the right decision for the drilling operation to mitigate all of the consequent problems^4^.

During the drilling operation, the power transmission to the drilling rig rotating system causes the drill bit rotation to make the hole and penetrate the drilled formation. The interaction between the drilled hole wall and drillstring, bottom hole assembly (BHA), and drill bite might cause some disturbance in the rotation mechanism and finally leads to unplanned drillstring downhole vibrations. The downhole vibrations for the drill string are represented by axial, torsional, and lateral vibrations^5^. The three types are different in the vibration action and the destructive nature of each mode. The axial vibration is represented by the vertical or longitudinal vibration mode and this might cause drill bit damage due to the bounce phenomena. The three vibration modes affect the drilling performance and equipment failure, however, the lateral mode has the least damaging impact^6^. The lateral type of downhole vibration characterizes the lateral motion from side to side and causes the eccentric rotation of the drillstring (whirl phenomena). The lateral vibration type is the most harmful vibration mode and can cause massive shocks when the bottom hole assembly hits the wellbore surface. The contact action between the wellbore and bottom hole assembly might push the system into a backward whirl in some situations^7^. Backward whirl is considered to be the utmost harmful type of vibration, actuating bending moment fluctuations with large magnitude, resulting in high rates of connection fatigue, damaging of bit cutter, and drilling an over-gauged wellbore. Forward whirl might be due to bending of the drillstring that happens due to imbalance in BHA and result in wear in BHA from one side. The third vibration mode is the torsional mode where the drillstring is restricted from rotation and this might cause the string to twist. In this type, the bite has a periodical rotation and stop cycles due to the rotation restriction. The bit stops (which is the stick action) for a certain time, and then the bit rotates (which is the slip action) due to the torque increase, and this process is known as stick–slip. The more severe the stick/slip, the longer the stuck period, and in sequence the higher the rotational accelerations. Torsional action causes fatigue in the bottom hole assembly connections and might damage bits. This vibration is usually expected in conditions such as deep wells and high-inclination long lateral wells^4^.

Many practical optimizations for the drilling parameters were proposed as solutions to mitigate the drillstring vibrations and consequent problems as drill bit design, utilizing the downhole mud motor, and providing optimum values for the controllable parameters as the weight on the drill bit, drilling rotation speed, drillability rate, rig torque, and pumping flow rate^5,8,9^. The drilling team has to take an effective mitigation approach for each downhole vibration scenario for enhancing drilling operation performance and reduce the possibility of adverse outcomes.

### Machine learning research for petroleum engineering

The petroleum industry is showing a great trend regarding machine learning applications, in addition, the main objective of the fourth industrial revolution for enhancing the digitization and automation of the industry encouraged to research for accomplishing many studies for machine learning applications in different disciplines and work scopes. The implementation of machine learning succeded to develop intelligent models for features prediction and operations performance optimization for cost and time savings in different scopes as the drilling performance prediction^10–12^, rheology estimation for fully automated mud monitoring systems^13–15^, predicting different rock petrophysical^16^ and geomechanical characteristics^17,18^, and well control domain for early kick detection^19–22^, in addition to other applications.

### Drillstring vibration prediction

A new novel trend toward implementing the surface rig sensor data and delivering the best value from such data for optimizing the drilling performance and mitigating the associated drilling problems was proposed in the literature and showed great outcomes in many studies^23–25^. It is common in the drilling operation to utilize the surface drilling data that is collected from the rig sensors to evaluate the drilling performance and manage the drilling process. This includes torque calculation, rotational speed, hook load, block position, flow rate, and many others. Traditionally, the drilling team uses surface torque and rotation speed to track drilling vibrations and in particular, to detect stick–slip. Several methodologies have been developed using only surface measurements or in combination with downhole measurements^26–28^.

Recently, many studies developed a variety of data-driven techniques for detecting downhole vibrations with machine learning techniques. Okoli et al.^1^ developed five ML models for classifying the downhole axial and lateral vibrations using the drilling data T, ROP, and WOB. The results showed that the Intra-BHA predicted downhole vibration severity from 50 to 80% accuracy level, while the Inter-BHA predictive accuracies were reduced significantly which was attributed to the different operating and hole conditions.

Zhao et al.^8^ presented a modified symbolic aggregate approximation to use drilling data to detect the drilling events anomalies for example stuck pipe, vibration, and fluid loss.

Baumgartner and van Oort^29^ utilize a machine learning technique to analyze and identify downhole vibrations using high-frequency downhole measurements collected by BHA-based sensors. Pollock et al.^30^ introduced a system with a neural network that simulates historical data implemented from the decisions of expert directional drillers with 3% or less error.

Zha and Pham^31^ developed a system to predict stick–slip in real-time from surface parameters only by training a deep neural network. The study used 1400 measurements data set for stick–slip classification from the drilling torque, tension, drilling speed, WOB, and triaxial acceleration data. The model showed an accuracy of 96% and 73% for model precision, while the model was further fed and trained that caused improving the performance with 99% validation accuracy of and 97% for model precision.

Ignova et al.^32^ developed a classification model using K-Means cluster analysis algorithm and principal component analysis (PCA) pattern recognition technique. The model was trained by 2048 measurements data set for the classification of good or damaged drilling using high-frequency shock data for the model training process.

Wiktorski et al.^33^ utilized the support vector machines (SVM) tool for developing two models one for detecting the stick–slip and the other model for the lateral vibration by training the models with 260 and 865 measurements for the stick–slip and lateral vibration models respectively. The stick–slip detection model used T, DSR, and WOB for model inputs and the results showed an accuracy of 98% coefficient of determination, while the lateral vibration model was trained using model inputs of T, DSR, and hook load with an accuracy of 93% coefficient of determination.

Gupta et al.^34^ employed the random forest and gradient boosting ANN for the stick–slip classification model using T, ROP, and WOB as model inputs. The overall model accuracy is 62% for detecting the stick–slip pattern. Another research was done to detect the three modes of vibrations in real time using an artificial neural network and the model evaluation showed great performance for prediction as the statistical metrics proved a higher coefficient of correlation (greater than 0.95) with low errors between actual and predicted values less than 4% average absolute percentage error^35^.

The developed models among the literature studied the topic from diverse aspects in terms of the ML approach, model input features, the data set measurements, output parameters, and model accuracy. Moreover, there is a shortage of research to study the three types of downhole vibrations (axial, lateral, and torsional) as an integrated modeling approach. Consequently, this study presents a new contribution toward the prediction of downhole vibrations using surface drilling data from rig sensors for auto-detecting the downhole vibration modes in real-time. The study developed different machine learning models [radial basis function (RBF), support vector machines (SVMs), adaptive neuro-fuzzy inference system (ANFIS), and functional networks (FN)] to evaluate the capabilities of these tools and get the best prediction results from the surface drilling data set. The models were trained using a horizontal section drilling operation. The outputs from this study will greatly add to the drilling operation automation to close monitor the vibrations and technically help the drilling team to take an efficient decision for mitigating the downhole vibration severity.

## Data and machine learning research approach

This section discusses in detail the research approach for developing the different models for vibration detection by sensor data through model optimization and evaluation for training, testing, and validation phases. Besides, integrated data description and analytics to explore the data interrelationship for the model features.

### Research approach

**Figure 1:**
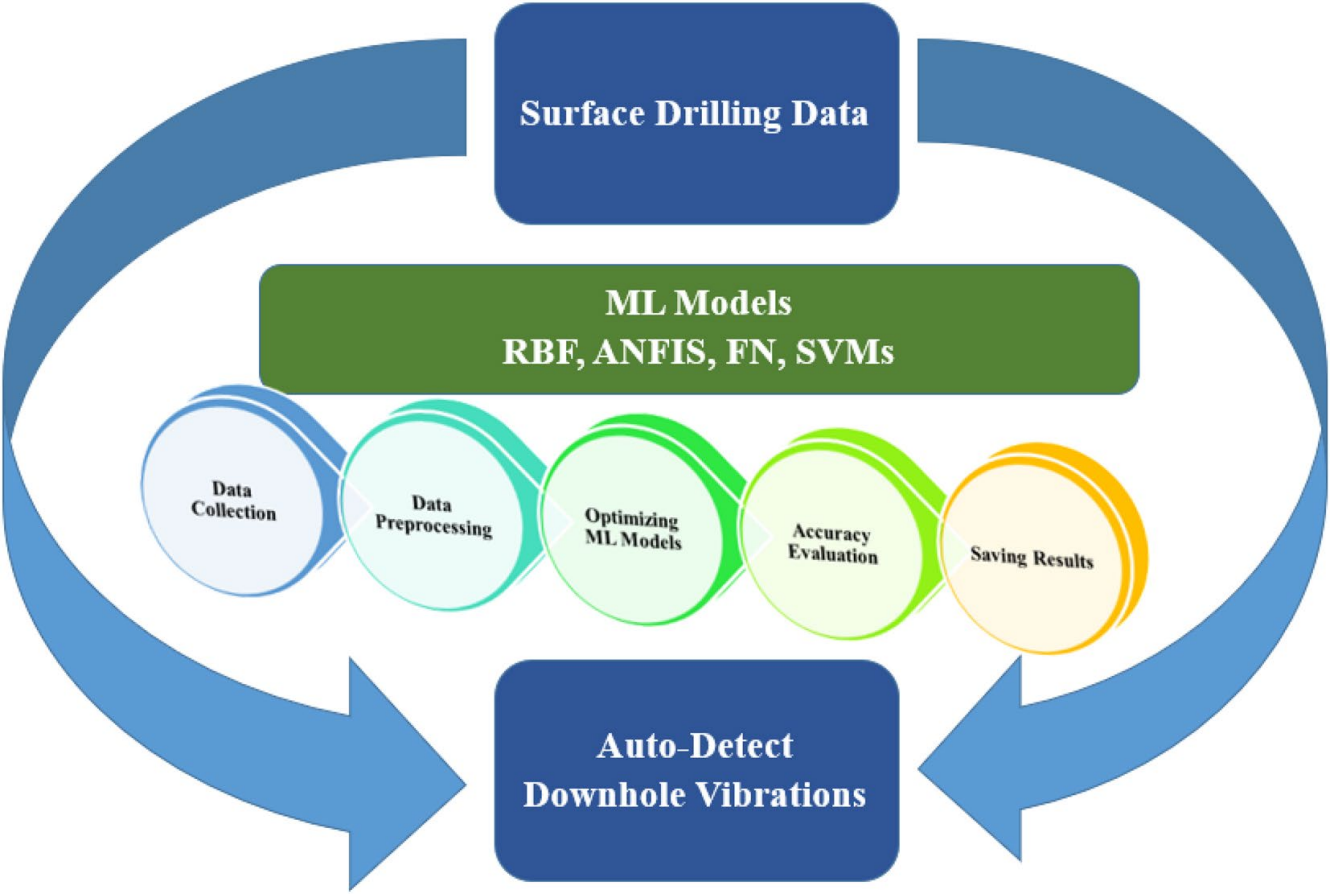
Study approach layout.

The study presented a technical approach that considered the full process for developing the ML models starting from collecting rig sensor data, data preprocessing, building the models, evaluating the accuracy, and finally saving the best results for vibration predictions (Fig. 1). The data was gathered from the rig sensors and then the data was forwarded to the data wrangling process for data cleaning and quality enhancement by removing the outliers and performing advanced statistical data analysis to study the data range, distribution, and correlation coefficient. Building the ML models started after the full data preprocessing phase and the model building was studied with deep sensitivity for the different learning algorithms and model parameters to provide the best results for the prediction of the downhole vibrations. The models were evaluated with statistical metrics to evaluate the performance and compare the predicted versus actual measurements for the downhole vibration values. The models would be retrained again with different training algorithms or model parameters in case the prediction performance was not accepted till reached the best accuracy level to report the best results and model parameters. The best prediction results from the optimized ML models were reported to show the models’ prediction capabilities.

### Data preprocessing

The current study utilized real field downhole vibration data during drilling horizontal sections to develop the different machine learning models. 5750 data points from a well data that show the surface drilling parameters (that represent the model input features) associated with the downhole vibrations measurements (that show the target parameters of the ML models). Mainly six rig sensors that record the drilling mechanical and hydraulic data are the basic inputs for the models’ named rate of penetration (ROP), drilling torque (T), drilling speed rotation (DSR), mud rate (Q), standpipe pumping pressure (SPP), and the weight on the drill bit (WOB), while the downhole vibrations were logged using downhole sensors for detecting the downhole drillstring vibrations (axial, lateral, and torsional vibrations). The developed models were further tested by a validation unseen data set (3950 data points) from a well data during drilling a similar horizontal section within the drilling phase.

The collected drilling data was preprocessed by diverse pipelines to remove the illogic values within the data set as zeros and negative values, remove the parameters outliers, and smooth the drilling data to remove the noise and enhance the data quality. The cleaned data set was then analyzed to explore the interrelationships and data distributions.

### Exploratory data analysis

The surface drilling parameters are commonly observed at the surface while drilling and these surface parameters are significantly affected by the interaction between the drillstring and wellbore wall which causes the downhole vibration. The application of statistical analysis to the data gathered demonstrates good variety and representation of the data as it encompasses a wide range of input and output parameters (Table 1).

**Table 1:** Statistical analysis for the models’ data set.

	Q	SPP	RPM	T	WOB	ROP	Lateral_Vib	Axial_Vib	Torsional_Vib
Mean	250.6	2666.3	118.2	11.1	21.2	66.6	3.9	0.7	1843.1
Std	10.8	196.8	1.3	0.4	1.9	12.9	1.4	0.2	143.0
Min	230.0	2279.4	108.5	7.3	10.4	43.7	2.7	0.5	1543.3
25%	239.1	2489.9	117.8	10.9	20.0	56.8	3.0	0.5	1734.8
50%	259.7	2658.5	118.4	11.1	20.6	63.9	3.3	0.6	1812.7
75%	260.2	2839.3	118.8	11.4	22.4	74.5	3.6	0.7	1929.2
Max	265.2	3003.5	120.8	12.2	25.2	106.9	7.2	1.2	2151.9

The pumping rate ranged from 230 to 265.2 ppg, the pumping rate from 2279 to 3003 psi, the drillstring rotation from 108 up to 120 rpm, the drilling torque from 7 up to 12 klbf.ft, the weight on bit from 10 to 25 klbf. and drillability rate from 43 to 106 ft/h. and the resulting vibrations show a range from 2.7 to 7.2 g, 0.5 up to 1.2 g, and 1543 to 2151 deg_per_s_sq for the lateral, axial, and torsional vibrations respectively.

**Figure 2:**
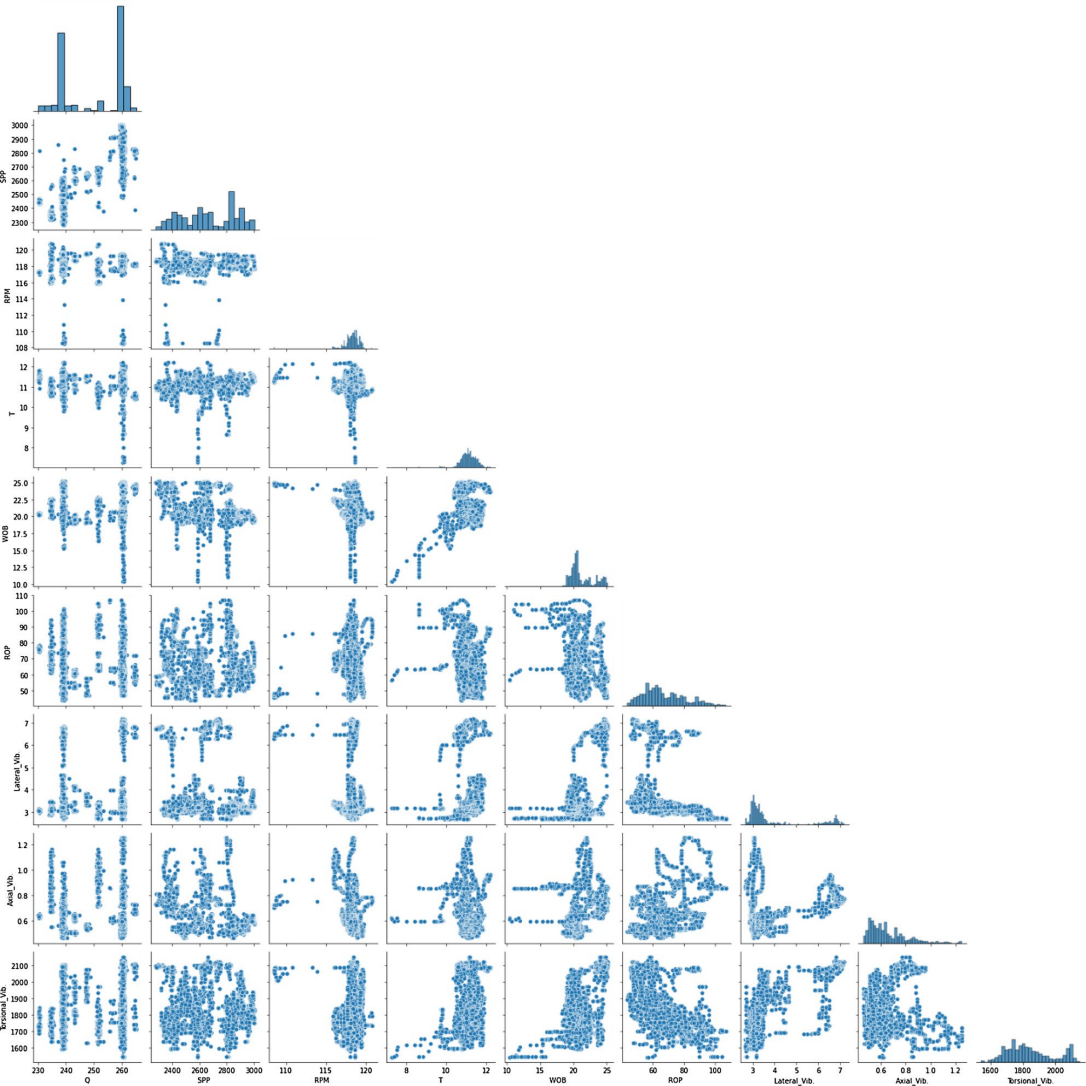
Pair plots for the data set features.

Figure 2 shows the pair plots for the data set features to represent the correlation of the parameters through visualization and it is clear from the plots that it is a complex nonlinear type of correlation between the drilling parameters and the three modes of downhole vibration.

**Figure 3:**
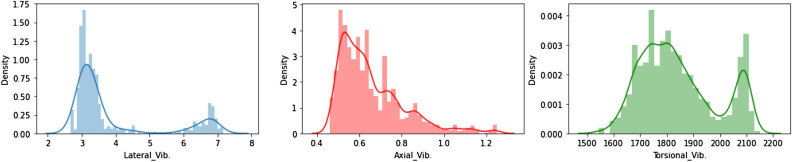
Vibration data distribution.

Besides, Fig. 3 displays the data distribution for the vibration data across the three modes of vibrations where lateral and torsional types showed bimodal distribution while the axial vibration showed positive right-skewed distribution.

**Figure 4:**
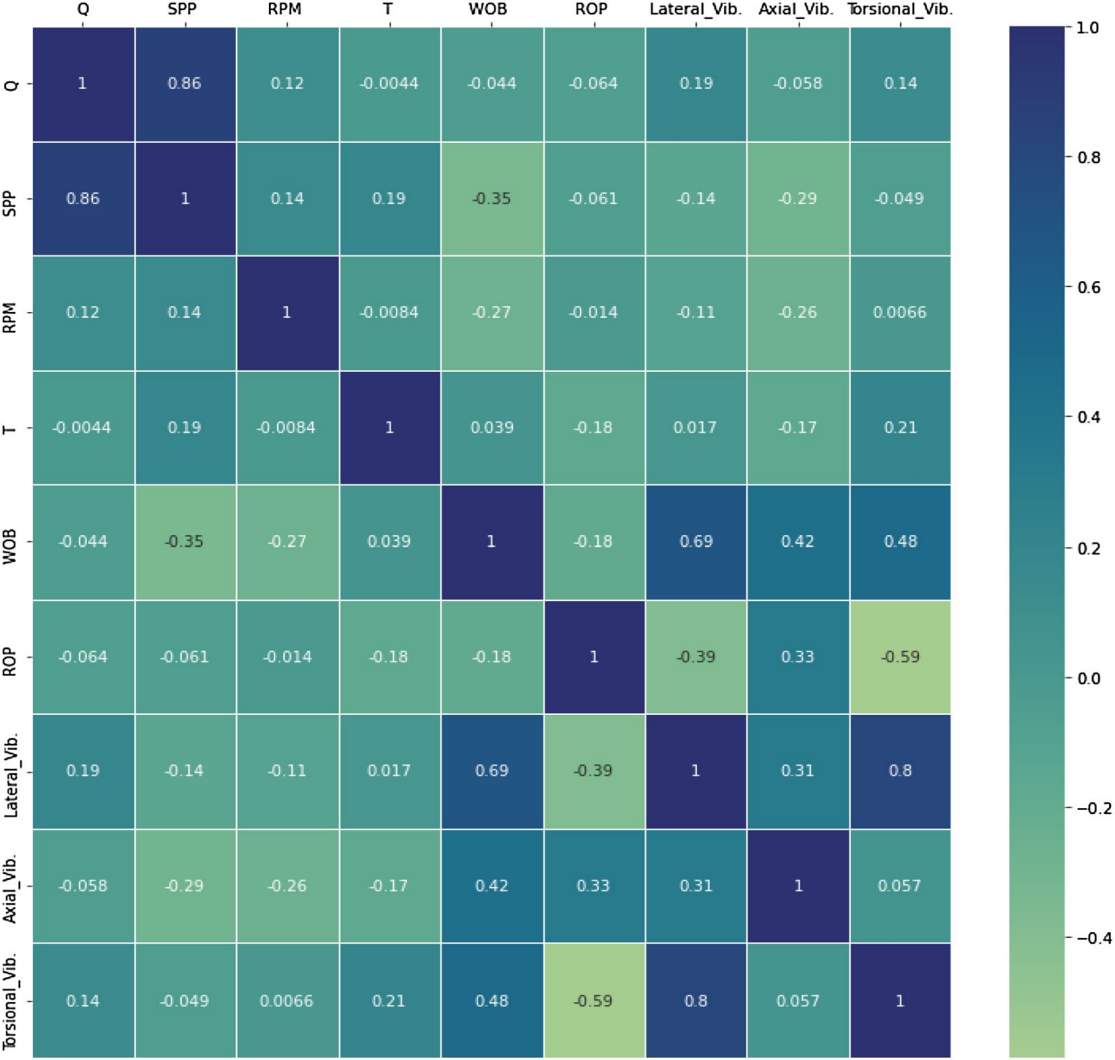
Data heat map (generated by Python https://www.python.org/).

In addition, the coefficient of correlation (R) between the model parameters was determined as shown from the heatmap in Fig. 4. The heatmap showed that the lateral vibration had a direct relationship with Q, T, WOB, axial and torsional vibration, while had an indirect relationship with SPP, RPM, and ROP with a variation in R-value. The axial vibration had an indirect relationship with all inputs except WOB and ROP which showed a direct relationship type. The torsional vibration data showed a direct relationship with all parameters except SPP and ROP. All three types of vibration data showed a direct relationship that had its maximum R-value between the lateral and torsional vibrations (0.8) while the minimum R was 0.057 between the axial and torsional vibrations. These statistics revealed the complexity between the data and might reveal nonlinear relationships between the parameters^36^, and therefore, the machine learning application for such problems will help to represent the intercorrelations between the parameters for predicting the downhole vibrations based on the learning capabilities from the model data and algorithms.

### Machine learning techniques

The study implemented four techniques to evaluate the capabilities of the algorithms to capture the parameter patterns for autodetecting the downhole vibrations from the surface drilling data. Radial basis function (RBF), support vector machines (SVMs), adaptive neuro-fuzzy inference system (ANFIS), and functional networks (FN) were utilized and compared during the training and testing for the models. These techniques are now widely applied in ML research applications for petroleum big data^37,38^.

The RBF is considered to have a simple architecture. Commonly, three layers for the model structure provide a simple way for the training exercise^39^. The RBF is found to be a great competent algorithm for modeling the data with a high degree of noise^40^. The technique has a non-linear type of transformation that is applied to the hidden layer weighting vector and provides a continuous function for multi-variable prediction by determining the best results for improving the accuracy^41,42^.

ANFIS is one of the ML tools that showed successful applications for solving technical problems with high computational capabilities for prediction purposes in oil and gas^13^. The tool is considered a modified model that comprises the neural networks and fuzzy interference systems^43^ by implementing diverse functions to every node for training the model features in fuzzy interference systems^44^. During the algorithm learning progression, changing the membership functions will be done to reach the best results for modeling the problem and this task is commonly controlled by Sugeno systems and Mamdani system as an optimization procedure^45^.

SVM is another ML technique that is used for classification problems and another application for regression purposes. The technique determines the weights and biases from the training data set to figure out the hyperplane which splits the maximum margin of the data^46^. The data with a high degree of complexity and dimensionality is found to be modeled by using the Kernel-type technique that has a function for non-linear high dimensional space^47,48^.

FN has an architecture of three layers that represent the input layer for the feed input parameters, the output layer for the model prediction, and the processing hidden layer that might be more than one single layer^49^. The neurons are connecting these layers and the output is calculated through the identified weights by the learning algorithms as forward or backward propagation types. Finally, the most optimum values for the output are determined^50^.

### Models evaluation

The developed models were evaluated during the sensitivity analysis for each model to get the best results for predicting the three models of downhole vibrations. The evaluation was achieved by calculating two statistical metrics as the coefficient of correlation and the average absolute percentage error (AAPE). R and AAPE are estimated between the real measurements for the vibration versus the predicted vibration values for every iteration through the model optimization phase. The two metrics were calculated as:

1 $$\mathrm{R}= \frac{\mathrm{N}\left(\sum_{1}^{\mathrm{N}}{\mathrm{Y}}_{\mathrm{i}}{\widehat{\mathrm{Y}}}_{\mathrm{i}}\right)-\left(\sum_{1}^{\mathrm{N}}{\mathrm{Y}}_{\mathrm{i}}\right)\left({\sum }_{1}^{\mathrm{N}}{\widehat{\mathrm{Y}}}_{\mathrm{i}}\right) }{\sqrt{\left[\mathrm{N }\sum_{1}^{\mathrm{N}}{\mathrm{Y}}_{\mathrm{i}}^{2}-{\left({\sum }_{1}^{\mathrm{N}}{\mathrm{Y}}_{\mathrm{i}}\right)}^{2}\right] \left[\mathrm{n}{\sum }_{1}^{\mathrm{N}}{\widehat{\mathrm{Y}}}_{\mathrm{i}}^{2}-{\left({\sum }_{1}^{\mathrm{N}}{\widehat{\mathrm{Y}}}_{\mathrm{i}}\right)}^{2}\right]}},$$

2 $$\mathrm{AAPE}=\left(\frac{1}{\mathrm{N}}\sum_{\mathrm{i}=1}^{\mathrm{N}}\left| \left.\frac{{\mathrm{y}}_{\mathrm{i}}-{\widehat{\mathrm{y}}}_{\mathrm{i}}}{{\mathrm{y}}_{\mathrm{i}}} \right|\right)\mathrm{ x }100\right.,$$

where N is the total number of points in the data set, ${\mathrm{y}}_{\mathrm{i}}$ represents the real measurement, $\hat{y}$_i_ is the model prediction, and $\overline{y}$ is the parameter mean.

## Results and discussion

Each model was tested during sensitivity analytics for a wide range of every parameter to get the optimized model parameters for the best accuracy and prediction results. This step is exactly performed by evaluating the model prediction accuracy in terms of R and AAPE through each case/run for the model. The optimized parameters for every model was saved and reported as a final phase.

### Models optimization

The model performance was determined through model training and testing for the data set. Table 2 presents the optimized parameters for every ML model that is achieved through deep sensitivity analysis across a wide range of parameters of each model. RBF was optimized by using 25 neurons for the network with Bayesian regulation backpropagation training algorithm and Hyperbolic tangent sigmoid transfer function. SVM model had the best results with 10 for kernel options and gaussian kernel function type. ANFIS model with 2 membership functions provided the highest accuracy among the iterations with gaussian type for the input membership function and linear type for the output membership function. FN with functional network forward–backward type and method of nonlinear of degree (2) showed the best results for vibration detection.

**Table 2:** Sensitivity analysis results for the optimized ML models’ parameters.

RBF		SVMs		ANFIS		FN	
Neurons no	25	Kernel option	10	Number of MFs	2	Type	Functional network forward–backward
Training function	Bayesian regulation backpropagation	C	100	InputMF	Gaussian	Method	Non-linear(2)
Transfer function	Hyperbolic tangent sigmoid	Lambda	1e-5	InputMF	LINEAR		
		Epsilon	1e-5				
		Verbose	1				
		Kernel	Gaussian				

It is worth mentioning that the developed models have the same structure for all three models of vibrations, not a separate model for detecting each vibration type and this is one of the challenges during the models' development.

### Models evaluation

Every ML model was trained using 4000 data points (70% of the model data set) and 1750 data points were utilized for testing the model that showed 30% of the total model data set. The next plots show the accuracy delivered by the models for auto-detecting the downhole vibrations in real time for training and testing the models.

**Figure 5:**
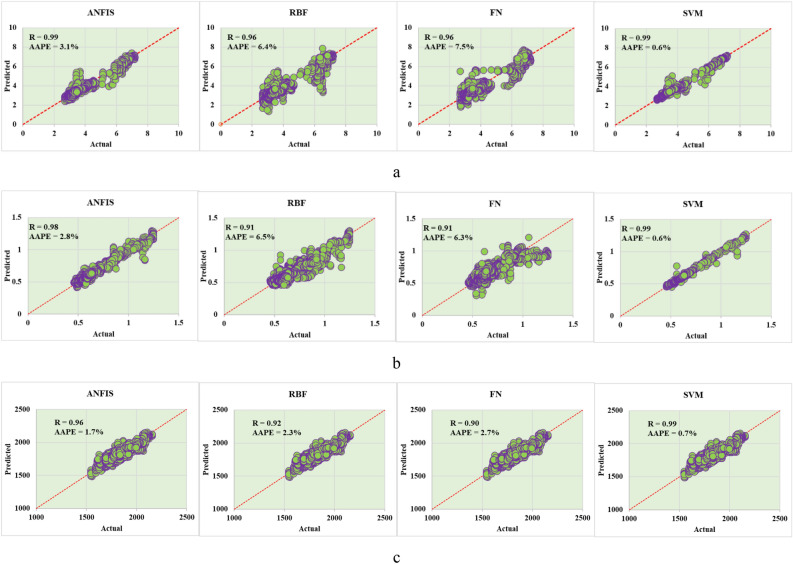
Training results for the developed vibration models (**a**) lateral (**b**) axial (**c**) torsional.

The training results for the developed vibration models are presented in Fig. 5 the best-case optimized models. The lateral vibrations show a high degree of prediction especially with SVM-model and ANFIS model and general prediction accuracy is higher than 0.9 for R and a maximum 7.5% AAPE. The other vibration models for the axial and torsional have the same trend with a high degree of match between actual and predicted values for the models' prediction.

**Figure 6:**
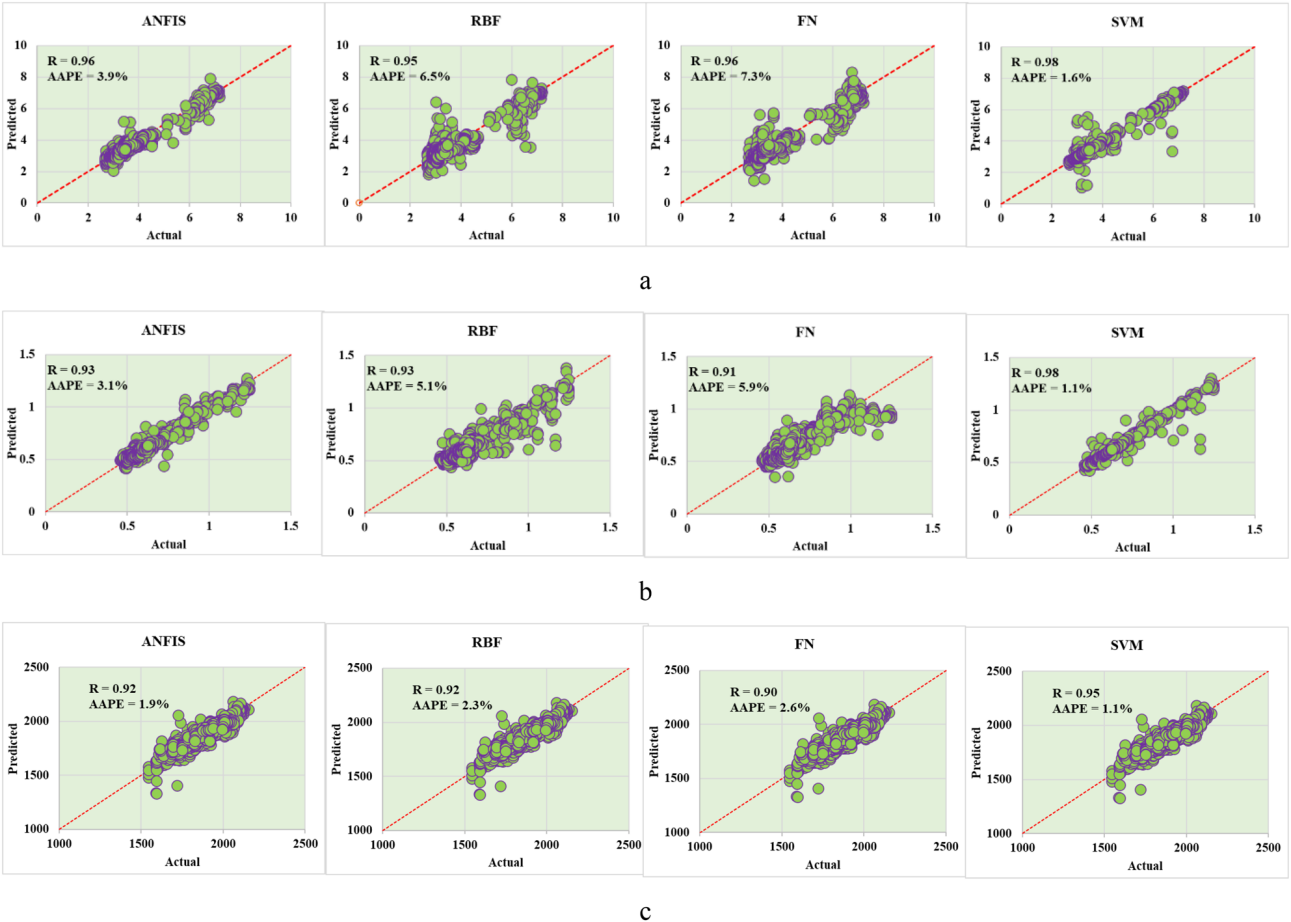
Testing results for the developed vibration models (**a**) lateral (**b**) axial (**c**) torsional.

Testing the developed models with the testing data set shows a high degree of accuracy for the vibration predictions as illustrated in Fig. 6. The accuracy for the developed models for the three types of vibrations showed that R ranged from 0.91 up to 0.98 with the best minimum AAPE of 1.1% and maximum value of 7.3%.

### Models validation phase

**Figure 7:**
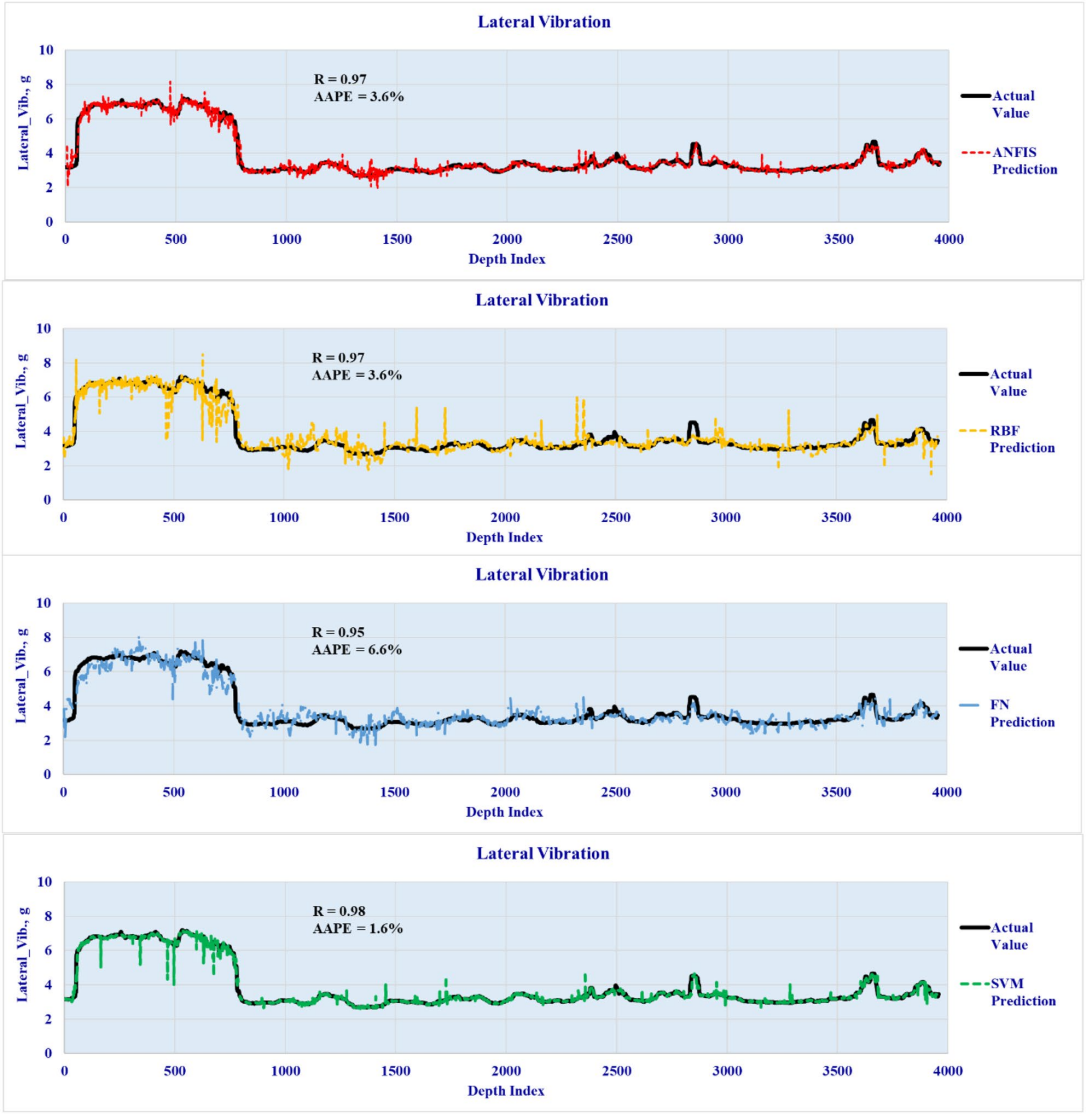
Lateral vibration prediction results (Validation well data set).

**Figure 8:**
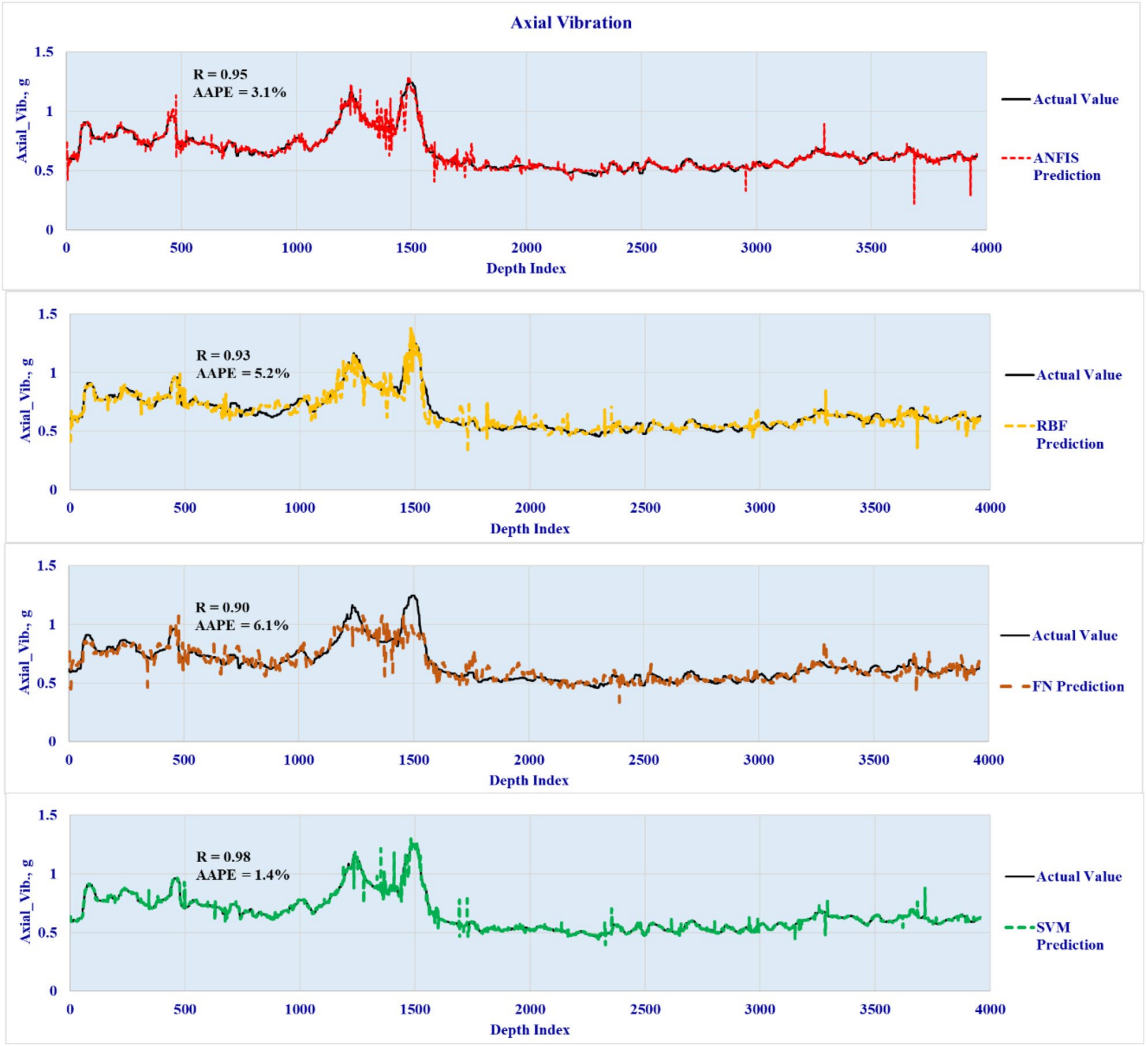
Axial vibration prediction results (Validation well data set).

**Figure 9:**
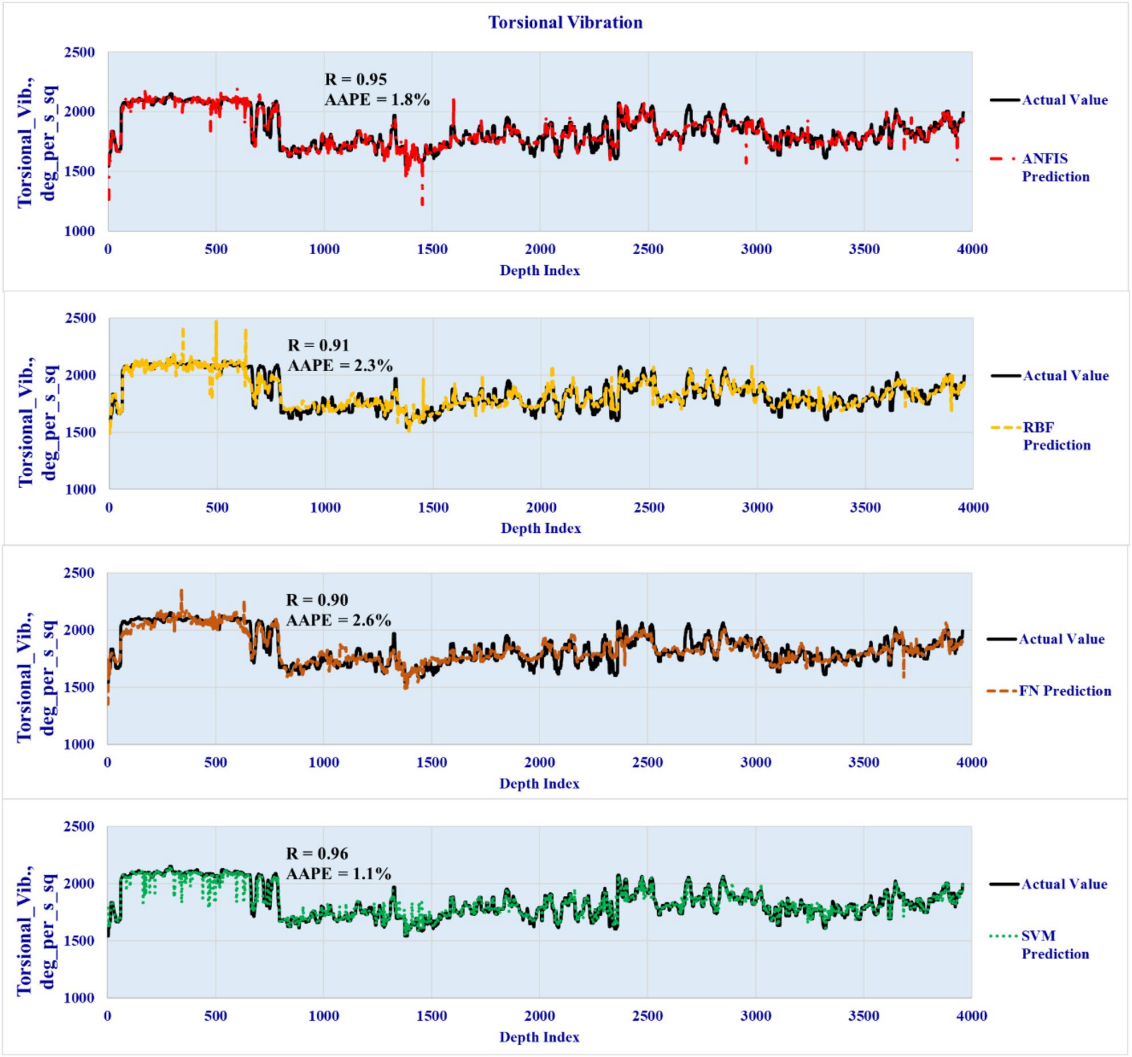
Torsional vibration prediction results (Validation well data set).

The four developed models were validated by testing the accuracy over unseen data set to predict the three vibration types in real-time using the well drilling data. The lateral validation results are plotted in Fig. 7 to highlight the predicted vibration profiles versus the actual downhole vibrations for the four developed models. The axial and torsional vibration predictions are presented in Figs. 8 and 9 respectively. The results showed a high degree of match with the actual downhole measurements for the three types of vibrations with R ranging from 0.9 to 0.98 and AAPE from 1.1 to 6.1%.

The real-time vibrations obtained from the developed ML models provide vital information on the durability of drilling BHA. This information is very helpful for directional drillers and drilling engineers. For directional drillers, it can help to identify vibrations and then apply correct mitigation for severe conditions and gives full control over the bottom hole assembly, also, tracking the vibrations trends obtained from the model it helps to avoid many tools failure by comparing predicted vibrations versus downhole tools limit such as red zone and continuing drilling without interruption to the well total depth especially while drilling horizontal sections. For drilling engineers, avoiding drilling interruption is required to minimize the non-productive time and in sequence more cost. In addition, giving good readings from downhole tools to be able to avoid deviations from the planned directional proposal especially in horizontal sections to be able to hit the targets as per well objectives.

## Conclusions

This research provided a technical approach for developing ML models for auto-detecting the downhole vibrations (lateral, axial, and torsional) in real-time based on the rig sensors’ surface data. The training data set was collected during the drilling horizontal section that comprised drilling data with downhole vibration measurements. The summary of this work can be concluded as follows:

- 5750 measurements data set was used to train four different techniques to predict the three modes of vibrations from drilling surface parameters named rate of penetration, drilling torque, drilling speed rotation, mud rate, standpipe pumping pressure, and the weight on the drill bit.
- The parameters optimization process of machine learning models was achieved through sensitivity analysis for each model`s parameters with performance evaluation using two statistical metrics R and AAPE.
- The optimized models showed strong prediction capabilities during the training and testing phases with overall accuracy for the three types of vibrations of R ranging from 0.91 up to 0.98 with a best minimum AAPE of 1.1% and maximum value of 7.3%. Besides, ANFIS and SVM models had the best-in-class accuracy performance.
- The developed ML models were validated with an unseen data set (3950 measurements well data) collected from the same field and environment to check the overall accuracy of the developed models that reported R ranged from 0.9 to 0.98 and AAPE from 1.1 to 6.1%.

The system proposed in this paper can be deployed on a drilling rig site to provide real-time monitoring of drilling dynamics and be a trigger for the automated advisory system, in addition, is helpful for directional drillers and drilling engineers for drilling optimization and better well planning. It is highly recommended to apply different advanced tools of machine learning through the most critical cases of downhole vibrations and through different well profiles to add more power for maximizing the drilling automation process.

## Data Availability

The datasets generated and/or analysed during the current study are not publicly available due to convedionality but are available from the corresponding author on reasonable request.

## Abbreviations

ML: Machine learning
RBF: Radial basis function
SVMs: Support vector machines
ANFIS: Adaptive neuro-fuzzy inference system
FN: Functional networks
BHA: Bottom hole assembly
AAPE: Average absolute percentage error
Q: Flow rate
R: Coefficient of correlation
ROP: Rate of penetration
RPM: Revolutions per minute
SPP: Standpipe pressure
T: Torque
WOB: Weight on bit
